# Vaccination in pregnancy

**DOI:** 10.3389/fgwh.2024.1523117

**Published:** 2025-01-13

**Authors:** Stephen H. Kennedy, Noni E. MacDonald, Sue Ann Costa Clemens

**Affiliations:** ^1^ Nuffield Department of Women’s and Reproductive Health, University of Oxford, Oxford, United Kingdom; ^2^ Department of Paediatrics, Dalhousie University, Halifax, NS, Canada; ^3^ Institute for Global Health, University of Siena, Siena, Italy; ^4^ Department of Paediatrics, University of Oxford, Oxford, United Kingdom

**Keywords:** vaccination hesitancy, pregnancy, COVID-19, maternal death, misinformation

## Perspective

During pregnancy, vaccine hesitancy, defined as a “delay in acceptance or refusal of vaccination, despite availability of vaccination services” (1) is understandable as mothers worry about possible effects on their unborn children. However, such concerns are exacerbated by widespread misinformation, as occurred during the COVID-19 pandemic. A 2024 systematic review has highlighted the extent to which social media platforms were disseminating untruths, e.g., vaccines are generally unsafe for pregnant woman and they increase the risk of infertility, miscarriage, stillbirth, and congenital defects (2). The false claim that COVID-19 vaccines result in female infertility (3) was not surprising given that the claim has repeatedly been made over many years about vaccines against polio and tetanus, and more recently human papilloma virus, especially in low-middle income countries (4).

Whatever the reasons for vaccine hesitancy, which include historical racism (5), the effects have been dramatic. In Europe, a 2024 literature review found that acceptance of vaccination against COVID-19 among pregnant women ranged from 21.3% to 87% and 29.5% to 82.7% for one and two vaccine doses, respectively (6). Given that vaccination protects against severe disease (7, 8), it was inevitable that some women would die unnecessarily if not vaccinated. In the UK, for example, COVID-19 was the second most common cause of maternal death in 2020–2022 contributing to the highest maternal mortality rate in 20 years (9). However, does the biomedical community bear any responsibility for the vulnerability of the pregnant population to these distortions of the truth?

At the outset of the COVID-19 pandemic the precise risks for pregnant women were uncertain, although it was known that SARS-COV-1 infection in pregnancy is associated with increased maternal mortality (10). Evidence began to emerge of similar risks for SARS-COV-2 infection from small case series and a living systematic review comparing pregnant and non-pregnant women with COVID-19 (11). Then, a cohort study involving 43 hospitals in 18 countries, showed from as early as 2 March 2020, significantly increased severe infections (relative risk (RR), 3.38; 95% CI, 1.63–7.01) and maternal mortality (RR, 22.3; 95% CI, 2.88–172) in pregnant women with COVID-19 compared to unaffected pregnant women (12).

Unfortunately, professional organizations and international bodies did not respond appropriately or quickly enough to the emerging evidence (13). In many countries, pregnant women were not included in the groups targeted for vaccination once vaccines became available; nor were they routinely offered vaccination after publication of the study in October 2021 comparing 10,861 vaccinated pregnant women matched to 10,861 unvaccinated pregnant controls that demonstrated the effectiveness of an mRNA vaccine (14). Concerns about the lack of clinical trial data, especially relating to the safety of the vaccines, led to vaccination guidelines for pregnant women across the world that were inconsistent and often contradictory.

In one sense, the chaos was predictable. Despite acknowledgment dating back to the 1990s that the interests of pregnant women were underrepresented in biomedical research (15), they were largely excluded from emergency vaccine trials initiated during the H1N1 influenza, Middle East Respiratory Syndrome Coronavirus (MERS-CoV), Zika, and Ebola outbreaks from 2009 to 2019 (16). A later study from 2018 to 2023 identified only 22/400 (6%) vaccine trials that included pregnant women (17). The recommendation to include pregnant women in such trials has a strong ethical basis that includes having access to research that provides the prospect of direct benefit to participants and their offspring (18).

A prime example of benefit to the unborn child is avoiding the life-long consequences of microcephaly resulting from infection with Zika virus (19). Hence, in 2016, the Wellcome Trust funded the PREVENT project (Pregnancy Research Ethics for Vaccines, Epidemics, and New Technologies), which initially focused on Zika virus. Its second report provided a “roadmap for the ethically responsible, socially just, and respectful inclusion of the interests of pregnant women in the development and deployment of vaccines against emerging pathogens” (20). The guidance aimed for the following: not unjustifiably exclude pregnant women from participating in vaccine studies; allow them and their offspring to benefit from advances in vaccine technologies, and give them access to safe and effective vaccines against emerging and re-emerging pathogen threats. One of the report's most important recommendations was to develop evidence-based strategies to promote confidence about vaccination in pregnancy among all stakeholders including women, their families, and, crucially, healthcare providers themselves. In this group of professionals, even the language of the package insert can have a negative effect on the perception of a vaccine's safety (21).

Progress has undoubtedly been made with regard to vaccination to protect newborns against influenza, tetanus, diphtheria and pertussis (Tdap), pneumococcus and respiratory syncytial virus (22), and potentially Group B streptococcus (23), all of which have been shown to be safe for both mother and baby, but it is not difficult to conclude that more could have been achieved globally since the PREVENT reports were first published.

In June 2024, Dr Anthony Fauci appeared before the US Select Subcommittee on the Coronavirus Pandemic and was held “publicly accountable” for showing “no remorse for the millions of lives affected by his divisive rhetoric and his unscientific policies” (24). Representative Marjorie Taylor Greene actually accused Dr Fauci of presiding over science that was “disgusting and evil” (25). This unprecedented personal attack is symptomatic of the continuing aggressive dissemination, at scale, of misinformation about vaccination. According to the Center for Countering Digital Hate (CCDH), the anti-vaxx industry is worth up to $1.1 billion to Big Tech with 62 million followers across their platforms (26). The issue is particularly relevant for pregnant women and their babies, who can be disproportionately affected by infectious diseases, as in the Zika, Lassa fever, and Ebola epidemics (27–29).

The barriers to vaccination uptake are considerable (30) and the interventions used to date have had limited success (31). When a scientist of Dr Fauci's stature is accused of falsehood, one realizes that the task of generating effectiveness and safety data and then convincing people that the data are genuine is massive. Matters are only likely to get worse if Robert F. Kennedy Jr. is appointed as US Secretary of Health and Human Sciences. However, as scientists we must continue to present the facts and in addition to developing new vaccines, we must implement data collection systems across pregnancy, birth, and early childhood using international standards (32, 33) to strengthen the argument that vaccination in pregnancy against emerging and re-emerging pathogens is essential when the benefits outweigh any possible risks.

## Data Availability

The original contributions presented in the study are included in the article/Supplementary Material, further inquiries can be directed to the corresponding author.
